# A structural equation model of factors associated with HIV risk behaviors and mental health among men who have sex with men in Malawi

**DOI:** 10.1186/s12879-020-05310-1

**Published:** 2020-08-10

**Authors:** Yuan Zhao, Amrita Rao, Andrea L. Wirtz, Eric Umar, Gift Trapence, Vincent Jumbe, Sosthenes Ketende, Dunker Kamba, Chris Beyrer, Stefan Baral

**Affiliations:** 1 Department of Epidemiology, Johns Hopkins Bloomberg School of Public Health, Baltimore, USA; 2 Center for Public Health and Human Rights, Johns Hopkins Bloomberg School of Public Health, Baltimore, USA; 3 Department of Health Systems and Policy Development, University of Malawi College of Medicine, Blantyre, Malawi; 4 Center for Development of People, Blantyre, Malawi; 5 Center for Global Health, Trinity College, Dublin, Ireland

**Keywords:** MSM, Sexual behavior stigma, Sexual risk behaviors, HIV, Structural equation model

## Abstract

**Background:**

Men who have sex with men (MSM) bear a disproportionate burden of HIV in Malawi. Early prevention efforts in Malawi have been largely focused on preventing heterosexual and vertical transmission of HIV, and MSM have rarely been the specific benefactors of these efforts, despite facing both higher prevalence of HIV coupled with multiple barriers to prevention and care. To better facilitate the design of culturally relevant HIV prevention programs and prioritize resources among MSM in resource limited settings, the objective of this analysis was to estimate the relationship between social factors and HIV related risk behaviors and mental health.

**Methods:**

338 MSM were recruited using respondent-driven sampling in Blantyre, Malawi from April 2011 to March 2012. Structural equation models were built to test the association between six latent factors: participation in social activities, social support, stigma and human rights violations, depression symptomatology, condom use, and sexual risk behaviors, including concurrent sexual partnerships and total number of partners.

**Results:**

The mean age of participants was 25 years old. Almost 50% (158/338) of the participants were unemployed and 11% (37/338) were married or cohabiting with women. More than 30% (120/338) of the participants reported sexual behavior stigma and 30% (102/338) reported depression symptomatology. Almost 50% (153/338) of the participants reported any kind of HIV-related risk behaviors and 30% (110/338) participated in one of the recorded social activities. Significant associations were identified between stigma and risk behaviors (β = 0.14, *p* = 0.03); stigma and depression symptomatology (β = 0.62, *p* = 0.01); participation in social activities and depression symptomatology (β = 0.17, *p =* 0.01).

**Conclusion:**

Results suggest MSM reporting stigma are more likely to report sexual risk practices associated with HIV/STI transmission and depressive symptoms, while those reporting participation in social activities related to HIV education are less likely to be depressed. Furthermore, interventions at the community level to support group empowerment and engagement may further reduce risks of HIV transmission and improve mental health outcomes. Taken together, these results suggest the potential additive benefits of mental health services integrated within comprehensive HIV prevention packages to optimize both HIV-related outcomes and general quality of life among MSM in Malawi.

## Background

The HIV epidemic is typically characterized as being generalized in Malawi, with 9.2% of adults 15–49 living with HIV in 2018 and an annual overall incidence rate of approximately 2.3% [1, 2]. Moreover, HIV remains the leading cause of mortality among adults in Malawi [1, 2]. In response to the HIV epidemic, the Malawian government has made substantial efforts to scale up HIV prevention and treatment, including expanding both voluntary HIV testing and counseling (HCT) and prevention of mother-to-child transmission services and promoting condom usage [3]. These efforts have been reflected by a steady decline of HIV prevalence among the adult population from 15.0% in 2001 to 9.2% in 2018 [1, 2].

As HIV prevention efforts in Malawi have largely focused on preventing heterosexual and vertical transmission of HIV, men who have sex with men (MSM) have rarely been the specific benefactors of these efforts, despite facing both a high burden of disease coupled with multiple barriers to HIV prevention and care [4, 5]. Previous studies suggest MSM in Malawi bear a disproportionate HIV burden with regional prevalence as high as 17.3% in 2017 [4, 5]. This burden is consistent with the historically limited HIV prevention programs specifically for MSM. In Malawi, limited clinical and cultural competency of health care staff for the specific health needs among MSM has also been identified as a significant barrier [4, 6, 7]. More broadly across Sub-Saharan Africa, data have demonstrated intersecting anticipated, perceived, and enacted stigmas limiting the coverage of available HIV testing and treatment [7]. With increased national efforts to reach the UNAIDS 90–90-90 targets, the Malawian National Strategic Plan for 2015–2020 began including specific approaches to address the high burden of HIV among key populations, specifically female sex workers and men who have sex with men (MSM). These efforts have included encouraging testing and providing community support for linkage to care and access to ART [3]. However, criminalization of same-sex practices has resulted in limited information about the HIV epidemiology and unmet needs among MSM. Few tailored healthcare programs exist to address the needs of the MSM community.

The lack of services is especially significant given the consistent body of evidence demonstrating that psychosocial challenges are prevalent among MSM and are associated with HIV risk behaviors [6, 7]. Lick et al. demonstrated that difficult social experiences, including antigay stigma and victimization, often leads to the experience of minority stress, which in turn contributes to disparities in physical health among sexual minorities [8]. Another widely accepted minority stress model proposed by Meyer indicates that minority stressors, such as victimization, concealment of sexual orientation and internalized homonegativity, have negative effects on mental health and that coping and social support can mitigate some of these effects [9]. However, studies of the association between other psychosocial factors, including depression and distress, and HIV risk behaviors and prevention practice among MSM have primarily been conducted in the US or other high-income countries. The present study in Malawi intends to provide insight into similar associations in resource-limited settings where same-sex relationships are highly stigmatized and criminalized. In particular, MSM who experience increasingly visible enacted stigma would face multiple barriers to accessing health services and social support. Quantitative measurement of sexual behavior stigma [10], social support and social activities would provide pertinent, in-depth information about the vulnerability and discrimination these men face and their urgent need of security and community support [11]. The results may also enable the development of new perspectives in the design of standard HIV prevention approaches among MSM [12].

To estimate the HIV prevalence and characterize associations between prevalent HIV and risk factors, a study conducted previously by Wirtz and colleagues between 2011 to 2014 assessed current HIV status and the barriers to seeking healthcare services among MSM in Blantyre, Malawi, particularly as they related to HIV and STI services [4]. The study remains one of the few studies specifically assessing the HIV related behavioral and psychosocial factors among MSM in Malawi [4]. As the legal and social environment of same sex practices has not changed significantly since 2014, a further analysis of the data would provide additional insight about the social determinants of risk behavior, mental health and stigma among MSM, supporting a more comprehensive prevention and treatment program to improve the wellbeing of those in the MSM community. Thus, to better facilitate the design of culturally relevant HIV prevention programs and prioritize resources among MSM in a resource limited setting, it was our aim to quantitatively estimate the relationship between social and mental health factors and HIV-related risk behaviors among MSM in Malawi.

## Methods

### Study design

The sample included 338 MSM who were recruited between August 2011 and March 2012 in Blantyre, Malawi via respondent-driven sampling (RDS). The details of RDS recruitment have been previously described [4]. Briefly, a total of 10 seeds were purposively selected and were each asked to invite three other participants to come in for the study [4]. Those participants who subsequently came in for the study were in turn asked to invite three additional members of their social network to participate [4]. This process continued for 19 waves of recruitment [4]. This cross-sectional survey served as the baseline study for the larger combination HIV prevention intervention (CHPI) cohort that aimed to test the feasibility of a comprehensive package of HIV prevention interventions [4]. Participation in the cross-sectional assessment included completion of an interviewer-administered survey and biological assessment of both HIV and syphilis. Eligibility criteria for participation included being 18 years or older, assigned male sex at birth, and fluent in Chichewa or English. Eligible individuals also had to report anal sex with another man in the last 12 months, report no prior participation in the study and provide informed verbal consent. The study was conducted in collaboration with both the Center for Development of People (CEDEP), a Malawi community-based organization that provided HIV prevention services to MSM, and the Malawi College of Medicine. The consent and enrollment processes were conducted in private rooms at the study office and staff were trained in confidentiality and human subjects protection. As reported elsewhere, crude HIV prevalence was 15.4% (RDS-weighted 12.5, 95% confidence interval (CI): 7.3–17.8) [4].

### Measures

The structured questionnaire consisted of seven modules designed to assess demographic and socioeconomic status, including age, employment and marital status, sexual behavior stigma, depression, knowledge, attitudes and behaviors; condom negotiation, sexual history, and social capital. As sexual behavior stigma and social support are diverse and complex constructs, which require relevant contextual and cultural information, we designed the questionnaire and measured those constructs based on formative research conducted in 2011 [13], instead of using standardized scales to capture the experience pertaining to local communities in Malawi.”

We utilized exploratory principal factor analysis to extract six latent constructs. We also calculated a Cronbach’s alpha for questions within each construct., including:

Outcome variables:

1. Condom usage and communication (α = 0.90): thirteen items measured condom usage. Example questions are “Do you insist on condom use with a long-time male partner?” and “How often do you use condom in the past 6 months?”
2. Sexual risk behaviors (α = 0.65): five indicators measured risk behavior, including concurrent sexual relationships, “Do you currently have two regular sexual partners?”, and number of partners, “Of the men you’ve had anal or oral sex with in the past 12 months, how many of them were main partner?” or “casual partners?”
3. Depression symptomatology: this construct consisted of only two indicators measuring the person’s mental status and included “Have you felt sad or had depressed mood for more than 2 weeks at a time in the last 3 years?” and “Have you ever felt like you wanted to end your life?”.

Exposure variables:

1. Sexual behavior stigma (α = 0.75): six items measuring MSM experiencing stigma related to sexual behavior, rejection from family or friends, or discrimination by healthcare providers. Examples are: “Have you ever felt excluded from family gatherings as a result of your sexual orientation or practice?”, “Have you ever been tortured as a result of your sexual orientation or practice?” and “Have you ever felt that you have received lower quality of health care as result of your sexual orientation or practice?”.
2. Social support (α = 0.78): five items measuring social support among MSM groups including questions such as “You can count on other MSM in your group of friends if you need to borrow money”, “You can count on other MSM if you need somewhere to stay” and “To help deal with a violent or difficult situation”.
3. Participation in social activities related to MSM health (α = 0.76): four indicators were summarized as a measure of engagement in community education activities about HIV and MSM health issues, sampled items include “In the past 12 months, how often have you participated in a meeting, march, rally or gathering to promote the rights of MSM?” and “In the past 12 months, how often have you joined together with other MSM to address a common problem facing MSM?”

### Data analysis

**Fig. 1:**
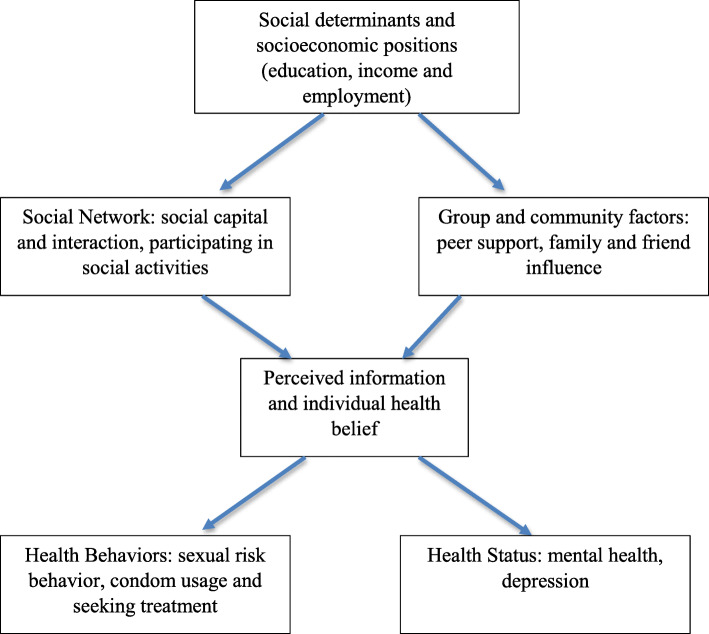
Hypothesized theoretical model of how social determinants influence risk behavior and mental health outcomes among MSM in Malawi

Several strategies were employed to explore the effect of RDS on distribution of key demographic and behavioral characteristics. As crude and RDS-weighted results were similar, for the convenience of model interpretation, we applied non-weighted (i.e. not weighted for RDS recruitment) structural equation modeling (SEM) for these analyses. Based on Meyer’s minority stress process model and Lick’s model of physical health disparities [8, 9, 14, 15], we created a hypothesized theoretical framework displayed in Fig. 1. This framework was used to evaluate the pathways of how social determinants, such as societal norms, stigma and social support, have a direct effect on individuals’ perception of sexual behavior and HIV risk prevention, which in turn affects sexual risk behavior, condom use and mental health status.

Initially, exploratory factor analyses were used to identify the measurement model of the latent constructs. Then a minor modification omitting the hierarchical relationship between socioeconomic position and social network factors was applied to the measurement model based on conceptual theory and factor loadings. To assess the internal consistency of indicators for each latent variable, Cronbach’s alphas were calculated.

Latent variables were treated as continuous to reflect their continuum of underlying dimensions in the population. Some of the measurement indicators for the latent variables were categorical or binary, including questions related to condom use and social support. As the measurement model reached satisfactory fitting, the structural model was specified based on our hypothesized theoretical framework using maximum likelihood estimation. The default full information generalized linear and mixed variable under GLLAMM framework [16, 17] and weighted least-square estimator were then applied to build the model.

To ensure the assumptions about missing data were reliable, a sensitivity analysis was performed using both multiple imputation and model-wise deletion to manage the missing data, and no substantive differences were identified. A diagnostic test was conducted to identify potential outliers and influential cases. No problematic output was found. The models were estimated using Mplus 7.11 (Muthén & Muthén, United States) and Stata 13 (StataCorp LP, TX).

### Model fit

The global model fit was evaluated following the theory proposed by Bollen and Long [18], in which several fitting indices were reported including Satorra-Bentler chi-square, the root mean square error of approximation (RMSEA), the comparative fit indices (CFI), and the goodness of fit statistic (GFI). Satisfactory global model fit is attained when RMSEA ≤ 0.05, CFI ≥ 0.95 and GFI ≥ 0.95, as shown in Table 4.

The mediation pathways were assessed based on global model fit statistics, Akaike information criterion (AIC) and Bayesian information criterion (BIC) fitting [19] or the coefficients of those pathways. To maximize the efficiency of the final model, insignificant paths were removed unless there was strong evidence of association from the theory or previous research. Based on multiple model fit statistics, including AIC and RMSEA, model 4 was selected as final model as it showed the best fit for our data.

## Results

### Participants

A total of 338 MSM were enrolled in the primary study via RDS [4]. Table 1 displays unweighted demographic characteristics. The mean age was 25.1 (SD = 5.2). Close to half of the participants (158/338) were unemployed and 11.0% were married or cohabiting with women (37/338). More than 30.0% (120/338) of the participants experienced rejection from friends and families because of their sexual orientation and almost 30.0% (102/338) reported symptoms of depression in the past 3 years. Almost 50% (178/338) of the participants reported having concurrent relationships in the last 12 months and 30% (110/338) participated in one of the recorded social activities.

**Table 1:** Demographic characteristics, stigma and depression symptoms of MSM in Blantyre, Malawi (*N* = 338)

**Variables**	**Mean**	**SD**
Demographic Information		
Age (year)	25.13	5.16
	**n**	**%**
Employment Status (*N* = include denominator for all variables, since some are missing data and do not total 338)		
Unemployed	158	46.75
Employed	136	40.22
Student	44	13.02
Marital Status (with women)		
Married or cohabiting	37	10.95
Single or divorced	301	89.05
Number of Children		
None	285	84.57
One or more	52	15.43
Lifetime experiences of stigma		
Family member(s) made a stigmatizing remark	82	24.33
Rejection by friends	118	35.01
Received a lower quality of healthcare	34	10.06
Tortured	50	14.88
Scared to walk in public	54	16.07
Beaten up/physically abused	40	11.83
Depression Symptomatology		
Felt sad for more than 2 weeks	102	30.18
Ever wanted to end one’s life	38	11.34
Having Concurrent relationships in last 12 months	178	52.67
Use condom with main male partner	119	63.00
Use condom with casual male partner	174	68.00

### Structural equation model

**Fig. 2:**
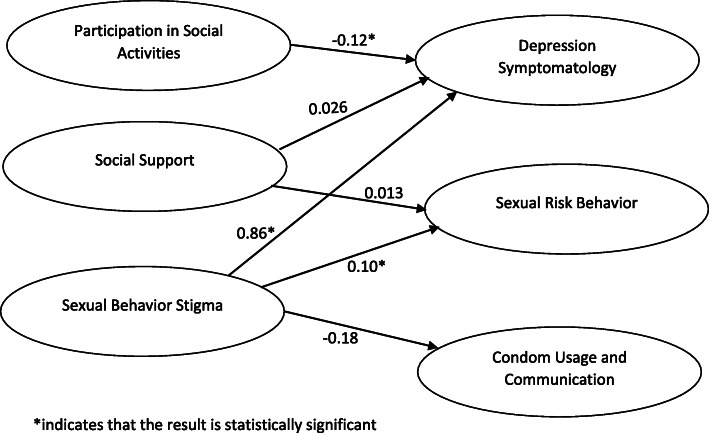
Structural equation model of relationship between latent variables raw coefficients among MSM in Malawi

Table 2 shows the measurement model of each latent construct and their corresponding measurement items. The factor loadings are presented in Table 2 and the fit of the modified measurement model met the criteria of adequate model fit. We fit four SEM models that each included the same six latent constructs and their corresponding measurement items. Model 1 included the pathways between the constructs as laid out in Fig. 2 adjusting for demographic variables of age, marital status and employment, model 2 adjusted for only significant demographic variables of age and marital status, model 3 included an additional pathway from depression symptomatology to sexual risk behavior to assess the potential mediation effect of depression symptomatology. Model 4 only included pathways in Fig. 2 without the demographic or mediation pathway. We selected model 4 as our final model based on the fitting index. Table 3 presents the relationship between latent structures with standardized estimates of the best-fit solution. These estimates can be interpreted as standardized regression coefficients; for example, an increase of one standard deviation in rejection was associated with an increase of 0.14 standard deviation of sexual risk behavior.

**Table 2:** Structural equation model results of measurements

**Constructs and Indicators**	**Estimate**	**S.E (*P*-Value)**	**95%CI**
Outcomes			
Factor 1: Condom Usage and communication (partial)			
Insist on condom use with male partner	1		
Insist on condom use with long-time partner	1.49	0.20(0.00)	(1.10,1.88)
Insist on condom use with regular male partner you trust	1.52	0.20(0.00)	(1.12,1.92)
How often using condom in the past 6 months	1.01	0.19(0.00)	(0.63,1.39)
Factor 2: Sexual Risk behavior			
Number of main partners	1		
Number of casual partners	4.36	2.56(0.09)	(−0.66,9.39)
Use the Internet for sexual partners	1.09	0.86(0.20)	(−0.60, 2.78)
Anytime concurrent partnerships	3.52	1.74(0.04)	(0.11,6.93)
Current concurrent partnerships	3.77	1.60(0.02)	(0.62,6.91)
Factor 3: Depression Symptomatology			
Have depressed mood for more than 2 weeks	1		
Ever wanted to end your life	0.32	0.11(0.00)	(0.10,0.53)
Exposures			
Factor 4: Sexual Behavior Stigma			
Excluded from family gatherings	1		
Family discriminatory remarks	1.18	0.19(0.00)	(0.81,1.55)
Lower quality of health care	0.69	0.13(0.00)	(0.43,0.95)
Ever been tortured	1.01	0.17(0.00)	(0.68,1.34)
Scared to walk around in public places	0.92	0.17(0.00)	(0.59,1.26)
Ever been beaten up	0.80	0.14(0.00)	(0.51,1.09)
Factor 5: Social Support			
Count on others to borrow money	1		
Count on others to the doctor	0.95	0.13(0.00)	(0.70,1.21)
Talk about problems	0.88	0.12(0.00)	(0.64,1.12)
Need places to stay	0.77	0.14(0.00)	(0.50,1.04)
Deal with a violent or difficult situation	0.89	0.13(0.00)	(0.63,1.15)
Factor 6: Participating social activities			
Promote the rights of MSM	1		
Speak with government officials or political leaders	0.68	0.12(0.00)	(0.46,0.91)
Address a common problem facing MSM	1.10	0.17(0.00)	(0.76,1.44)
Participated in an HIV prevention organization	1.12	0.19(0.00)	(0.84,1.59)

**Table 3:** Structural model of latent constructs

**Constructs**	**Standardized Estimate**	**SE (*P-*Value)**	**95% CI**
Condom Usage and Communication ON			
Age	−0.14*	0.057(0.01)	(−0.025, −0.027)
Sexual Behavior Stigma	−0. 086	0.065(0.18)	(− 0.021, 0.041)
Sexual Risk Behavior ON			
Age	−0.13*	0.062(0.04)	(−0.25, − 0.005)
Marital Status	−0.019	0.064(0.77)	(−0.14, 0.11)
Sexual Behavior Stigma	0.14*	0.067(0.03)	(0.008, 0.27)
Social support	0.034	0.06(0.62)	(−0.10, 0.17)
Depression Symptomatology ON			
Sexual Behavior Stigma	0.62*	0.07(0.00)	(0.47, 0.76)
Social Support	0.034	0.07(0.63)	(−0.10, 0.17)
Participating Social Activities	−0.17*	0.07(0.01)	(0.31, −0.03)

* indicates results are statistically significant (p < 0.05)

Figure 2 illustrates the same association as Table 3 between constructs in the non-standardized estimate. Sexual behavior stigma, especially from family, were associated with increased sexual risk behaviors (β = 0.14, *p* = 0.03) and depression symptomatology (β = 0.62, *p* < 0.01), but negatively associated with condom use and communication within partnerships (β = − 0.09, *p* = 0.18). Past participation in social activities concerning MSM health was associated with reduced depression symptomatology (β = − 0.17, *p* = 0.01). There was no significant association between social support and risk behavior (β = 0.03, *p* = 0.62) or depression (β = 0.03, *p* = 0.63). Model 3 also suggests depression could be an important mediator between sexual behavior stigmas and risk behavior.

The fitting indices of the proposed model are reported in Table 4. Model 4 used in the final results suggests adequate fitting considering the complex model, with (χ^2^(556) =1626.27, *p* < 0.001; CFI = 0.85; RMSEA< 0.05). Satisfactory model fit suggests the hypothesized association between these constructs is reasonable. Sensitivity analysis was performed using both multiple imputation and model-wise deletion strategies. There was no substantive difference in magnitude nor directionality in the results identified using these two statistical methods for handling missing data, though confidence intervals were much wider in the model-wise deletion method.

**Table 4:** Sensitivity analysis and fitting of different models^a^

	AIC	BIC	RMSEA	CFI	TLI	Likelihood Ratio
Model 1^b^	28,375.563	28,834.33	0.042	0.82	0.892	1721.48
Model 2^c^	28,374.800	28,829.74	0.042	0.83	0.892	1722.72
Model 3^d^	25,211.71	25,636.06	0.046	0.83	0.896	1625.80
Model 4^e^	25,208.17	25,624.88	0.045	0.85	0.898	1626.27

^a^: all 4 models have the same measurement model; changes of pathways were only applied to structural models

^b^: Model 1 is original model with all the proposed association as Fig. 2 and demographic variables of age, marital status, employment, etc

^c^: Model 2 is the model with only significant demographic variables, namely, age and marital status

^d^: Model 3 is the model with all significant demographic variable and significant latent association, with additional association of depression symptoms to sexual risk behavior

^e^: Model 4 is the model used in our results, with association indicated in Fig. 2

## Discussion

This study assessed the relationship between social and mental health factors and HIV-related risk behaviors among a group of marginalized cisgender men who have sex with men disproportionately affected by HIV in Malawi. Strong and significant associations were identified between sexual behavior stigma and increased HIV and STI-related sexual behaviors. Moreover, additional associations were noted between stigma and symptoms of depression. Finally, the value of resilience mediated by social capital was demonstrated by the inverse associations between past participation in social activities supporting MSM and depression symptomatology.

The findings presented here are consistent with previous studies in countries across sub-Saharan Africa, which suggest health outcomes of social stigmas including experiencing stigmas from family and community among MSM can lead to seeking acceptance and intimacy through not using condoms with sexual partners [20, 21]. Structural stigmas including criminalization of same-sex practices in Malawi likely potentiates both the burden and impact of individual stigmas ultimately serve as barriers to education and communication about safer sex among men. This same discrimination may challenge novel ART-based prevention and treatment strategies including pre-exposure prophylaxis and universal treatment for those living with HIV. These results suggest the added value of the implementation of culturally-relevant HIV prevention programs which include training for clinical providers to improve sociocultural tailored care for MSM to increase the quality and coverage of care as well as inclusion of community leadership in the design and implementation of HIV research and programs to build resilience and mitigate stigmas [20].

Moreover, the findings of prevalent depression symptomology among MSM are consistent with minority stress affecting men who have sex with men. Minority stress is likely exacerbated by sociocultural stressors as noted by Lick and colleagues where demonstrated discriminatory social policies and rejection of healthcare access can lead to cognitive changes including perceptual vigilance and rejection sensitivity [8, 21]. These cognitive changes have been associated with mental health stressors including depression and anxiety among lesbian, gay and bisexual individuals [22].

Notably, participation in community activities supporting MSM, including those connected to reducing stigma and raise HIV awareness may serve to increase resilience through social capital and mitigate some of the effects of depression [20]. And while cross-sectional, elements of resilience were also reproduced in analyses with significant associations seen between social support and lower symptoms of depression. In this study, there was no significant relationship between social support and HIV-related sexual risk behaviors which is also noted in other cross-sectional studies by Grover et al. [23] and Brown et al. [24] among MSM in Eswatini, however it is inconsistent with previous reports from Pantalone et al. [25], which employed a longitudinal design. One possible explanation is that our instruments only assessed the social support from MSM peers, and in highly stigmatizing environments, social capital from a small group of peers may be of limited population level mental and sexual health benefit. However, taken together, these data raise awareness of mental health stress affecting MSM in Malawi with more than one in three reporting at least one symptom. In the current HIV response, there is limited integration of effective mental health interventions. While mental health is critical to quality of life, it is also likely important in supporting linkage and retention in evidence-based interventions directed toward HIV prevention, treatment, and care.

### Limitations

The results presented here should be interpreted in the light of several limitations. First, the analytic process of extracting latent constructs from several survey domains needs to be carefully evaluated. As the primary goal of the parent study was to assess HIV prevalence among MSM in Malawi and related risk factors, the measures were intended to be brief to reduce confusion and burden for participants. Therefore, some indicators, for example, those measuring depression symptomatology are drawn from standardized measures [26]. Although internal consistency tests were performed to validate those indicators, further validation may be needed to apply those measurements in this specific population.

Second, the cross-sectional design makes it difficult to draw any causal inference. In our analysis, rigorous statistical models are used and all potential confounders of demographic characteristics are examined, but we still cannot rule out the possibility that other extraneous variables may have affected the estimated relationship between exogenous and endogenous variables and between endogenous variables themselves. Additional challenges arise when there is possibility of causality acting in a reciprocal manner, for example, the association between participation in social activities and depression could also act in the opposite direction, as we may expect individuals who are more depressed to be less active in social activities. Those concerns can only be solved with longitudinal data. Importantly, the data presented here were collected from 2011 and 2012. Given the ongoing criminalization of same-sex practices, limited scale of specific HIV prevention and treatment programs, and limited epidemiologic data among MSM in Malawi, these data remain relevant. Finally, SEM usually requires stricter linearity assumptions about how covariates are related to each other and to the outcomes [27]. SEM generally makes such assumptions to gain statistical power, which is useful to explore a wide range of different pathways across an entire set of variables, but caution is needed in interpreting the association as a causal relationship. However, SEM remains a powerful tool for exploratory analysis and for the hypothesis-generating process. Future evaluation using longitudinal data, and testing the same model in other settings would be informative.

## Conclusions

Currently, most HIV prevention interventions are reaching MSM on the individual level with a focus on behavioral interventions but a limited emphasis on intersecting stigmas that affect individual level risks. The data presented here quantitatively reinforce the need to move beyond individual level interventions to address structural and social determinants including stigma that are likely more actionable and ultimately more impactful in decreasing the risk of HIV acquisition and transmission risks. Moreover, these data also suggest that more effective integration of mental health interventions is critical given minority stress affecting MSM, including cognitive behavioral therapy and potentially even pharmaceutical interventions. The data presented on intersecting stigmas reinforce the need to include not only MSM as part of these interventions, but also the communities and societies in which they live. Standardizing and quantifying levels of multiple forms of stigma can also serve as the basis for evaluating interventions mitigating that stigma. And while it may be complex to implement and evaluate stigma mitigation interventions, it is also likely that these interventions are fundamental to the ultimate impact of interventions to decrease HIV incidence among MSM, including PrEP and universal treatment.

## Data Availability

The datasets used and/or analysed during the current study are available from the corresponding author on reasonable request.

## Abbreviations

AIC: Akaike information criterion
ART: Antiretroviral therapy
BIC: Bayesian information criterion
CEDEP: Center for Development of People
CFI: Comparative fit indices
CHPI: Combination HIV prevention intervention cohort
GFI: Goodness of fit statistic
HCT: HIV testing and counseling
HIV: Human immunodeficiency virus
MSM: Men who have sex with men
RDS: Respondent-driven sampling
RMSEA: Root mean square error of approximation
SEM: Structural equation modeling
STI: Sexually transmitted infection
